# Transgelin-2 is upregulated on activated B-cells and expressed in hyperplastic follicles in lupus erythematosus patients

**DOI:** 10.1371/journal.pone.0184738

**Published:** 2017-09-14

**Authors:** Kaori Kiso, Hajime Yoshifuji, Takuma Oku, Masaki Hikida, Koji Kitagori, Yoshitaka Hirayama, Toshiki Nakajima, Hironori Haga, Tatsuaki Tsuruyama, Aya Miyagawa-Hayashino

**Affiliations:** 1 Center for Anatomical, Pathological and Forensic Medical Research, Graduate School of Medicine, Kyoto University, Kyoto, Japan; 2 Department of Rheumatology and Clinical Immunology, Graduate School of Medicine, Kyoto University, Kyoto, Japan; 3 Center for Innovation in Immunoregulative Technology and Therapeutics, Graduate School of Medicine, Kyoto University, Kyoto, Japan; 4 Research Portfolio & Science, Drug Discovery Research, Astellas Pharma Inc, Tsukuba, Japan; 5 Laboratory for Molecular Cell Physiology, Department of Life Science, Akita University; 6 Department of Diagnostic Pathology, Kyoto University Hospital, Kyoto, Japan; Instituto Nacional de Ciencias Medicas y Nutricion Salvador Zubiran, MEXICO

## Abstract

Transgelin-2 (TAGLN2) is an actin-binding protein that controls actin stability and promotes T cell activation. TAGLN2 is also expressed on B-cells but its function in B-cells is unknown. We found that TAGLN2-expressing B-cells were localized in the germinal center (GC) of secondary lymphoid tissues and *TAGLN2* mRNA was significantly upregulated after IgM+IgG stimulation in primary human B-cells, suggesting that TAGLN2 was upregulated upon B-cell activation. In support of this, lymph nodes (LNs) from patients with systemic lupus erythematosus (SLE), in which the intense GC activity have been recognized, showed increased TAGLN2 expression in B-cells compared to control LNs. Moreover, TAGLN2^+^B-cells were distributed widely not only in the GC but also in the perifollicular areas in SLE LNs. In contrast, CD19^+^ B-cells and CD19^+^CD27^+^ memory-B cells in peripheral blood of SLE patients showed no increase in TAGLN2 mRNA. Two-photon excitation microscopy of Raji cells demonstrated that TAGLN2 colocalized with F-actin and moved together to the periphery upon stimulation. *TAGLN2*-knockdown in Raji cells resulted in impaired phosphorylation of PLCγ2 leading to inhibition of cell migration. Microarray analysis of *TAGLN2*-knockdown Raji cells showed decreased expression of the genes associated with immune function including *CCR6* and as well as of those associated with regulation of the actin cytoskeleton including *ABI2*, compared to controls. These results suggest that TAGLN2 might regulate activation and migration of B-cells, in particular, the entry of activated B-cells into the follicle. We also suggest that TAGLN2 could be used as a marker for activated B-cells.

## Introduction

Transgelin-2 (TAGLN2) is a member of the calponin family and has been characterized as a smooth muscle cytoskeletal protein. It may participate in the regulation of actin cytoskeleton dynamics [1]. The TAGLN family comprises three isoforms, and TAGLN2 is the only TAGLN present in leukocytes [2]. TAGLN2 is upregulated in colorectal cancer [3] and lung adenocarcinoma patient tissue samples [4] and may be involved in tumor development [5]. TAGLN2 expression is down-regulated in Barrett’s adenocarcinoma patient tissue samples [6]. TAGLN2 has not been reported in association with inflammatory diseases.

TAGLN2 in T-cells is critical for the regulation of T-cell activation through actin-mediated intracellular activation signaling and stabilization of the immunological synapse [2]. TAGLN2 has been found to be overexpressed in B-cell lines (Raji and MEC1) compared with myeloid and T-cell lines, which suggests that this protein may play a role in B-cell development [7]. Proteomic analysis indicated that TAGLN2 expression in peritoneal B-1 cells is over 60-fold that in splenic B-2 cells [8]. TAGLN2 expression is inducible in splenic B-2 cells, since TAGLN2 expression in B-2 cells was up-regulated to levels similar to those found in peritoneal B-1 cells by stimulation with mitogenic stimuli such as IgM and LPS for 48 h [8]. Cross-linking of surface IgM leads to association of the B-cell receptor (BCR) complex and tyrosine kinases with the cytoskeletal matrix and actin polymerization plays an essential role in B-cell activation [8]. Thus, TAGLN2 may be related to B-cell activation. A recent report showed that a deficiency of TAGLN2 (TAGLN2^-/-^) in B-cells has little effect on B-cell development and activation [9]; however, the role of TAGLN2 in B-cells is not well understood. High expression of TAGLN2 in B-1 cells suggests that TAGLN2 may be involved in immune diseases [8]. Systemic lupus erythematosus (SLE) is a prototypical autoimmune disease and is known to be associated with polyclonal B-cell hyperreactivity [10,11]. Reactive follicular hyperplasia is considered to be the most frequent finding in the lymph nodes in SLE patients, which reflects intense germinal center (GC) activity [12]. Thus, in this study, the clinical relevance of TAGLN2 was examined in B-cells in SLE patients. Since secondary lymphoid organs (e.g., spleen and lymph node) other than the kidneys or skin are rarely studied in SLE patients [10,11], we examined lymph nodes associated with SLE to validate the TAGLN2 expression in B-cells in SLE.

## Materials and methods

The pathology database in our institution contains 5 archives of biopsied formalin-fixed, paraffin-embedded lymph nodes from SLE patients with lymphadenopathy. Control lymph nodes that were biopsied to rule out malignancy and which turned out to be negative for tumor (n = 5), tonsils for tonsillectomy (n = 5), renal biopsies of lupus nephritis, class IV (n = 7), and kidney tissues from non-tumor areas taken at the time of resection for renal cell carcinoma (n = 5) were selected. The samples were taken at Kyoto University Hospital during the period from 2006 to 2015 and were stored in formalin- fixed paraffin-embedded blocks.

### Immunofluorescent analysis of tissue samples

Double immunofluorescence staining of tissues was performed with anti-TAGLN2 (Thermo Fisher Scientific, Yokohama, Japan), and anti-CD20 (Clone L26, DakoCytomation, Glostrup, Denmark) or anti-CCR6 (GeneTex Inc. Irvine, CA, USA) antibodies and the signals were detected with the Opal 2-Plex Kit, Cyanine 5/Fluorescein (PerkinElmer, Inc. Waltham, MA, USA). Cell nuclei were visualized with DAPI (Dojindo, Kumamoto, Japan). Fluorescence imaging analysis was performed using the FSX100 Fluorescence Microscope (Olympus, Tokyo, Japan). The number of TAGLN2^+^CD20^+^ cells in approximately a thousand CD20^+^ cells was counted in each section, and the average number of positive cells was calculated in SLE patients’ tissues and controls.

### *TAGLN2* mRNA expression in peripheral blood B-cells

Peripheral blood was obtained from consenting 17 SLE patients and 12 healthy donors. Human peripheral blood mononuclear cells (PBMCs) were isolated from the blood using Lymphocyte Separation Solution (Nakalai Tesque, Kyoto, Japan). CD19^+^B-cells were isolated from PBMCs using MACS Pan B Cell Isolation Kit (Miltenyi Biotec, Bergisch Gladbach, Germany). Subpopulation of CD19^+^CD27^+^ (BD Pharmingen, Tokyo, Japan) memory B-cells were sorted using a flow cytometer (FACSAria, BD Biosciences, San Jose, CA). CD38 expression (BD Pharmingen) as a B-cell activation marker was examined in CD19^+^ B-cells and CD19^+^CD27^+^ memory B-cells. cDNA was synthesized using SuperScript III 1st strand cDNA Synthesis System for reverse transcription-PCR (Life Technologies, CA, USA). Quantitative real-time PCR (qRT-PCR) reactions were performed in 384-well plate with TaqMan gene probes and primers designed by Life Technologies (CA, USA) for *TAGLN2* (assay ID: Hs00761239_s1) and *ACTB* (assay ID: Hs01060665_gl). These reactions were performed on an Applied Biosystems ViiA 7 real time PCR system with the TaqMan Fast Advanced Master Mix (Life Technologies, CA, USA). mRNA expression was normalized to *ACTB*, using the 2-ΔΔCt method.

### Primary human B-cell simulation

The blood was obtained from 6 healthy donors. CD19^+^B-cells were isolated from PBMCs using EasySep Human B Cell Enrichment Kit (STEMCELL Technologies, Vancouver, BC, Canada). Purified B-cells (1x10^5 cells) were maintained in 96-well cell culture plate in RPMI 1640 medium supplemented with 10% FBS (Invitrogen), 40 ng/mL IL-4 (PeproTech Inc., NJ, USA) and 10 μg/mL CD40L (PeproTech Inc., NJ, USA) in the presence or absence of anti-human IgM+IgG (eBioscience, San Diego, CA, USA). Total RNA was extracted from cell pellets using the RNAqueous-Micro Total RNA Isolation Kit (Thermo Fisher Scientific, MA, USA) and were incubated for 1, 6 and 24 hours. qRT-PCR reactions were performed in 384-well plates with TaqMan gene probes and primers designed by Life Technologies for *TAGLN2* and three reference genes, RPS18 (assay ID: Hs01375212_g1), RPLP0 (assay ID: Hs00420895_gH) and YWHAZ (assay ID: Hs01122445_g1). These reactions were performed as described above. TAGLN2 mRNA expression was normalized to the mean of three reference genes using the 2-ΔΔCt method. Data are presented as fold change relative to expression levels of non-stimulated controls.

### Patient consent and confidentiality

All sample collection and use of clinical records were performed under the written consent of study participants, and the study was conducted according to the principles expressed in the Declaration of Helsinki. The Ethics Committee of Kyoto University approved this study (Nos. R0305-1, G520).

### RNA interference

Raji B-cells (RCB3673) were provided by the RIKEN BioResource Center (Tsukuba, Ibaraki, Japan) through the National Bio-Resource Project of the MEXT, Japan and were maintained in RPMI 1640 medium supplemented with 10% FBS (Gibco, Thermo Fisher Scientific). Transient transfection of Raji cells was performed using the Amaxa Cell Line Nucleofector Kit V (Lonza, Basel, Switzerland). Cells (2 x 10^6) were resuspended with siRNA targeting *TAGLN2* (SR305508; 3 unique 27-mer siRNA duplexes; OriGene Technologies, Rockville, MD, USA) or a scrambled negative control siRNA in 100 μL of electroporation buffer, followed by electroporation with the Nucleofector™ 2b Device (Lonza).

### Immunoblotting

Raji cells were lysed in ice-cold RIPA Buffer (Nakalai tesque, Kyoto, Japan) for 1 h on ice. Cell lysates were centrifuged at 10,000 *g* for 10 min at 4°C and the supernatant was eluted with SDS sample buffer and heated for 5 min. The proteins were separated through 10–12% SDS-PAGE gels and transferred to a nitrocellulose membrane (BIO-RAD, Hercules, CA, USA). The membrane was blotted with antibodies against the following proteins: TAGLN2, PLCγ2 (CST, Danvers, MA, USA), phospho-PLCγ2 (Tyr759) (CST), Akt1/2 (Santa Cruz Biotechnology, Dallas, TX, USA), phospho-Akt (Ser473) (CST), p-ERK (Tyr204) (Santa Cruz Biotechnology), p44/42 MAPK (Erk1/2) (CST), PI3 kinase p85α (CST), phospho-PI3 kinase p85(Tyr485)/p55(Tyr199) (CST) and beta-actin (Abcam, Cambridge, UK). All antibodies used were anti-human rabbit polyclonal antibodies except for the anti-p-ERK and anti-beta-actin antibodies, which were anti-human mouse monoclonal antibodies. Goat anti-rabbit or mouse IgG-HRP (Santa Cruz Biotechnology) were used as secondary antibodies. The signals were visualized with Chemi-Lumi One Super (Nakarai tesque, Kyoto, Japan), and images were obtained using Ez-Capture MG (Daihan Scientific Co., Ltd., Gangwon-do, South Korea). Visualized bands were analyzed using the CS Analyzer (Atto Corporation, Tokyo, Japan).

### Migration assay

Since Raji cells express CXCR5 [13], which plays a role in B-cell migration, we assayed the migration of a cell suspension of Raji cells transduced with *TAGLN2*-specific siRNA (OriGene) or a scrambled negative control siRNA for 24 h. The cells, suspended in RPMI1640 containing 1% FBS, were loaded into the upper chamber of the CytoSelect™ 24-Well Cell Migration Assay (Cell Biolabs, Inc. San Diego, CA, USA) and were cultured for 4 h. Cells migrating into the lower chamber, which contained RPMI1640 plus 1% FBS with 750 ng/mL of recombinant CXCL13 (CXCR5 ligand; Biolegend, San Diego, CA, USA) were lysed and quantified using the CyQuant GR Fluorescent Dye and a fluorescence plate reader at 480 nm/520 nm (Infinite F200, TECAN, Tecan Japan, Kanagawa).

### Detection of cell surface proteins

For the analysis of surface CXCR5 and IgM expression levels, siRNA transfected cells were recovered and stained with FITC-labelled anti-CXCR5 antibody (Miltenyi Biotec, Bergisch Gladbach, Germany) and biotin-labelled anti-IgM antibody (Abcam) in combination with streptavidin-APC (eBioscience) according to the manufacturers’ instructions. Stained cells were then analyzed by a flow cytometer (FACSCalibur, BD Biosciences, San Jose, CA, USA).

### Intravital imaging of Raji cells in vitro

Raji cells were transiently transfected with 2 μg of an expression plasmid encoding GFP-tagged TAGLN2 (OriGene) and LifeAct-TagRFP (ibidi, Martinsried, Germany) using the Amaxa Cell Line Nucleofector Kit V (Lonza). For the preparation of cell images, cells 24 h after transfection were placed on a 35-mm glass dish with medium containing liquid collagen (Cellmatrix type 1-A, Nitta Gelatin Inc.), RPMI 1640, HEPES, NaHCO3, and NaOH (5 x 10^6 cells/ mL). In these experiments, the cells were stimulated with 10 μg/ mL F(ab’) anti-human IgM + IgG (eBioscience). Imaging data were acquired using an Incubator Fluorescence Microscope equipped with a multi-photon laser scanning system (LCV110 and FV1200MPE, Olympus) with a 25 x 1.05 NA water-immersion objective (XLPLNWMP, Olympus) and FV-10ASW software (Olympus). Fluorescence excitation was performed with a pulsed laser (InSight™ DeepSee™, Spectra Physics). The cells were kept at 37°C in 5% CO2 during the experiments. XYZT scanning data were acquired at 1.0-μm intervals in the Z-dimension and by Free Run in the T-dimension. Figures were processed using an open source Java image processing program, ImageJ (https://imagej.net/Welcome).

### Microarray analysis

Total RNA was isolated from Raji cells using the RNeasy Mini Kit (QIAGEN, Tokyo, Japan) following the manufacturer’s instructions. Amplified sense-strand cDNAs were generated, fragmented and labeled using the GeneChip® WT PLUS Reagent Kit (Thermo Fisher Scientific K.K., Kanagawa, Japan), and then were hybridized to the Affimetrix® Gene chip® Human Gene 2.0 ST Array (Thermo Fisher Scientific K.K.) for 16 h at 45°C according to the manufacturer’s instructions. The hybridized arrays were washed and scanned using Gene chip® Scanner 30007G. Miroarray data was analyzed to identify genes whose expression differed by <0.75-fold from the control. These differently expressed genes were classified according to their gene annotation for pathway analysis using the software, Microarray Data Analysis Tool Ver3.2. (Filgen, Inc., Aichi, Japan). To validate the microarray analysis data, qRT-PCR was performed to check the expression of some genes, using the following forward and reverse primers:

CCR6 forward, 5’-GAA GGG AGT GGA TCA GAG CA-3’;
CCR6 reverse, 5’-GTA TGG TTC AGC CCC TTC AG-3’;
ABI2 forward, 5’-TAT CCA GGC ATC CCA GCT AC-3’;
ABI2 reverse, 5’-ACC AAT TTC TCT TCT TGC AAC TTT-3’;
MLCK3 forward, 5’-TTT GAA GGG CTC TCG GAG G-3’;
MLCK3 reverse, 5’-CCA CTC GTG TTT CAG GCA C-3’;
PPP3R2 forward, 5’-GAT GTG CTC CCA CTT TGA CA-3’;
PPP3R2 reverse, 5’-CGT AAA TGC TGA ACG CAA AC-3’.

### Statistical analysis

Statistical analyses were performed using Graphpad Prism 6 (MDF Co., Ltd., Tokyo, Japan). Unpaired and Paired Student’s *t*-test, or the Mann-Whitney U test were used. Data are presented as the means with standard error of the mean (SEM). A p-value <0.05 was considered to be statistically significant.

## Results

### TAGLN2 is expressed on B-cells in the GC and *TAGLN2* mRNA in B-cells is induced upon BCR stimulation

**Fig 1:**
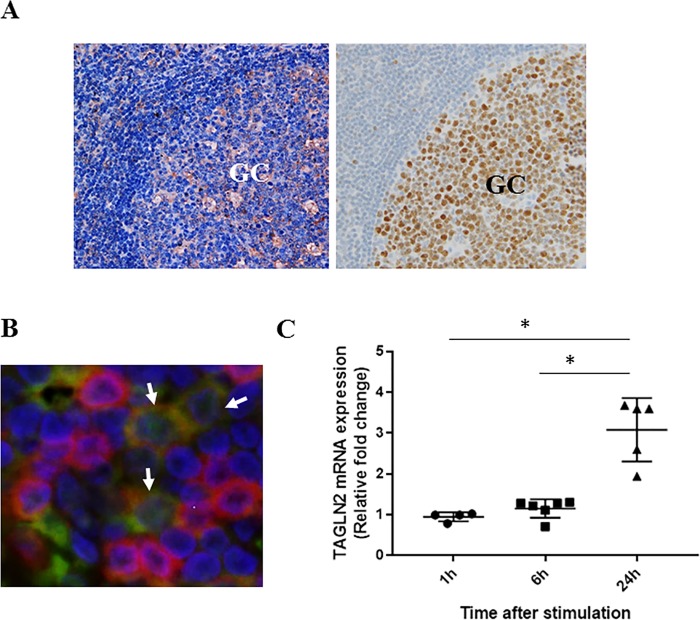
*TAGLN2* mRNA is induced upon BCR stimulation and significantly expressed on activated B-cells in the germinal center. (A) Immunohistochemistry showing TAGLN2 localized in the germinal center in human secondary lymphoid tissues (left) and Bcl-6 expression, a marker of germinal center B-cells (right) (original magnification, x400). GC, germinal center. The figure is representative of 5 tonsils examined. (B) Double immunofluorescence analysis of TAGLN2 (green) and the B-cell marker CD20 (red), showing coexpression of TAGLN2 and CD20 in the germinal center. Nuclei were stained with DAPI (arrows, original magnification, x400). (C) *TAGLN2* mRNA is induced upon BCR stimulation. *TAGLN2* mRNA levels were measured in primary human B-cells from six different healthy donors at the indicated time points after IgM+IgG stimulation using qRT-PCR. At each time point, the *TAGLN2* mRNA levels in anti-IgM+IgG stimulated cells (stim) are expressed fold change relative to the levels in non-stimulated (non-stim) cells. The mean *TAGLN2* mRNA expression ratio was 0.93-fold at 1 h, 1.09-fold at 6 h, and 2.85-fold at 24 h after stimulation, suggesting that *TAGLN2* mRNA was induced by 24-h B-cell activation (p<0.05, paired t-test vs. non-stimulated control). Data are presented as the mean ± SEM. *p<0.05.

TAGLN2^+^ cells were observed in the GC in human tonsils, shown as transcription factor Bcl-6-positive areas (Fig 1A) [14], and in paracortical T-cell areas. TAGLN2 was co-localized with CD20^+^ B-cells in GC (Fig 1B). Since the histological observation suggested that TAGLN2 may be induced on activated B cells, primary B-cells from healthy donors were stimulated with IgM+IgG. Fig 1C shows *TAGLN2* mRNA expression, which is indicated as the ratio of its expression in stimulated B-cells to in non-stimulated B-cells, at the indicated time points. The mean of this *TAGLN2* mRNA expression ratio was 0.93-fold at 1 h, 1.09-fold at 6 h, and 2.85-fold at 24 h after stimulation, suggesting that *TAGLN2* mRNA was induced by 24 h B-cell activation (p<0.05, paired t-test vs. non-stimulated control). These data suggested that TAGLN2 was highly expressed on activated B-cells after BCR stimulation in GC in secondary lymphoid tissues.

### A large number of TAGLN2^+^ B-cells are observed in lymph nodes and kidneys of SLE patients

**Fig 2:**
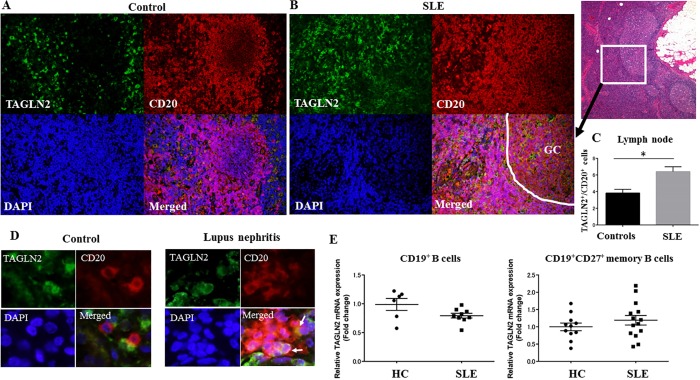
A larger number of TAGLN2^+^ B-cells is observed in lymph nodes and kidneys in SLE patients compared to those in controls. (A-B) Double immunofluorescence analysis of TAGLN2 and the B-cell marker CD20 is shown. Nuclei were stained with DAPI. (A) In control lymph nodes, TAGLN2^+^ B-cells were observed in follicular areas (original magnification, x200). (B) TAGLN2^+^B-cells were distributed from the follicular/germinal center (GC) to perifollicular areas in SLE lymphadenopathy (original magnification, x200). HE staining on the left (original magnification, x40). (C) The ratio of TAGLN2+ cells to CD20+ cells in SLE and controls was calculated. Significantly higher numbers of TAGLN2-positive cells were seen in B-cells in lymph nodes associated with SLE (n = 5) compared to those in controls (n = 5). Data are presented as the mean ± SEM. *p<0.05. (D) Right, a few TAGLN2^+^ CD20^+^ B-cells (arrows) are seen within B-cell aggregates in the interstitium of lupus nephritis, whereas no TAGLN2^+^ B-cells are observed in the control kidney sample (Left). (original magnification, x400). (E) Left, *TAGLN2* mRNA levels in CD19^+^ B-cells in healthy donors (HC, n = 6) and SLE patients (n = 9) as determined by qRT-PCR (mean ± SEM, 0.98 ± 0.10 in HC and 0.79 ± 0.04 in SLE) (p>0.05). Right, *TAGLN2* mRNA levels in CD19^+^CD27^+^ memory B-cells in healthy donors (HC, n = 11) and SLE patients (n = 14) (the mean of SEM, 1.0 ± 0.11 in HC and 1.2 ± 0.14 in SLE) (p>0.05.).

Since an intense GC activity has been recognized in SLE lymphadenopathy [12], the clinical relevance of TAGLN2 was examined in lymph node samples and peripheral blood B-cells in SLE patients. TAGLN2^+^B-cells in SLE lymphadenopathy were distributed in follicular areas as well as widely outside of the follicular areas, suggesting that TAGLN2^+^B-cells included GC B-cells and post-GC B-cells including memory B-cells. In contrast, in control lymph nodes, TAGLN2^+^B-cells were observed only in follicular areas (Fig 2A and 2B). Upon quantification, there was a significantly higher number of TAGLN2^+^B-cells in SLE than in controls (p<0.05) (Fig 2C). In lupus nephritis (n = 7), 12 ± 3% (the mean ± SEM) of CD20^+^B-cells (214 ± 134 cells) in the interstitium showed TAGLN2 expression (Fig 2D). The control kidneys (n = 5) showed minimal inflammation and a few B-cells (1 ± 0.63 cells) were observed, which were negative for TAGLN2 (both for numbers of B-cells and TAGLN2^+^ B-cells, lupus nephritis versus control, p<0.05).

We examined TAGLN2 mRNA expression of peripheral blood CD19^+^B-cells and CD19^+^ CD27^+^ memory B-cells isolated from SLE patients and healthy donors. There was no significant difference in the mRNA expression of *TAGLN2* both in CD19^+^ B-cells (n = 9) and CD19^+^CD27^+^ memory B-cells (n = 14) isolated from the peripheral blood of SLE patients compared to those from healthy donors (n = 6 and 11, respectively) (p>0.05) (Fig 2E). To assess B-cell activation state, CD38 expression was assessed by flow cytometry and the proportions of CD38^+^ cells among CD19^+^ B-cells or CD19^+^CD27^+^ memory B-cells were calculated (S1 Fig). There was a statistically significant increase in CD38^+^ cells among CD19^+^CD27^+^ memory B-cells in SLE patients compared to those in controls (p<0.05). CD38^+^ cells also tended to be increased among CD19^+^ B-cells in SLE patients compared to those in controls (p = 0.05). The details of the relative *TAGLN2* mRNA expression levels and the ratio of CD38^+^ cells among CD19^+^ B-cells or CD19^+^CD27^+^ B-cells in SLE patients and controls are given in S1 Table.

### TAGLN2 moves together with F-actin upon BCR stimulation

**Fig 3:**
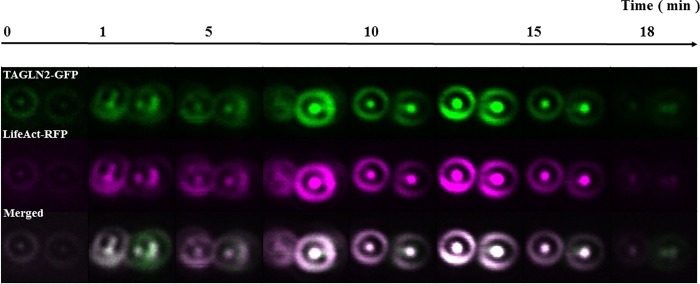
TAGLN2 colocalizes with F-actin and moves together to the periphery after stimulation. TAGLN2-GFP overlaps with LifeAct-RFP in Raji B cells at the outer actin ring. Raji cells co-transfected with TAGLN2-GFP and the actin probe LifeAct-RFP were stimulated with IgM+IgG, and the localization of each protein was then followed over the next 18 min using time lapse fluorescence microscopy. Top: TAGLN2-GFP (Green), middle: LifeAct-RFP (Magenta), bottom: merged image. The images marked as 0 were acquired before stimulation. Both the actin and TAGLN2 signals increased in intensity and thickness after IgM+IgG stimulation and were depleted from the central area of the cells and moved together to the periphery.

Next we examined the movement of TAGLN2 using live cell imaging of TAGLN2 and F-actin during B-cell activation. TAGLN2 co-localized with F-actin circumferential rings during BCR activation in Raji cells. Raji cells that were transiently transfected with the F-actin binding Life-Act-RFP probe and GFP-tagged TAGLN2 were stimulated with IgM+IgG and the cellular localization of these proteins was analyzed over 18 min of stimulation using immunofluorescence. The raw image data were processed by a Gaussian filter. Representative images at different time points are shown in Fig 3. Concatenated images taken before and after stimulation are shown in S1 Video and S2 Video, respectively. The circumferential rings which have been reported to be a rearrangement of F-actin in B-cells [15] were observed to increase in thickness from time 0 up to approximately 13 min after stimulation and subsequently to get thinner, based on the intensity of the RFP signal. TAGLN2-GFP co-localized with these F-actin circumferential rings and the GFP signal intensity increased and decreased along with the changes in the RFP signal intensity.

### *TAGLN2* knockdown leads to diminished phosphorylated PLCγ2, which is related to actin-linked signaling pathways

**Fig 4:**
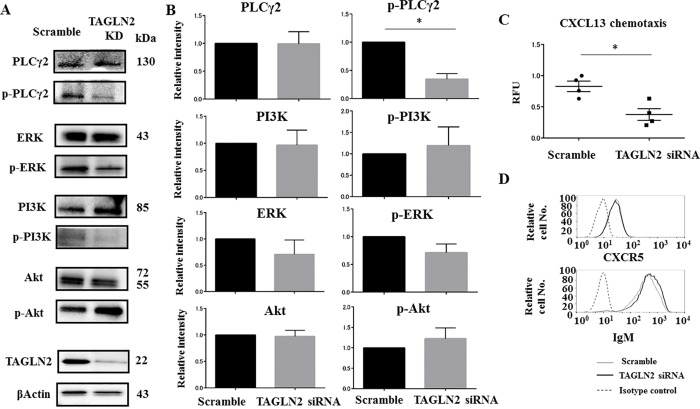
*TAGLN2* knockdown results in impaired phosphorylation of PLCγ, which is related to the actin-linked signaling pathway and inhibition of cell migration. (A) The total protein levels of PLCγ2, PI3K, ERK, and Akt and the levels of their phosphorylated forms after 10 min IgM+IgG stimulation of *TAGLN2*-knockdown (KD) Raji cells transfected with TAGLN2 siRNA, or of control cells with scrambled siRNA (Scramble), were detected by immunoblotting. Actin was blotted as a loading control. Suppression of TAGLN2 protein expression and PLCγ2 phosphorylation was seen in the *TAGLN2*-KD Raji cells compared with the scrambled siRNA transfected cells. (B) Normalized expression levels of each protein and each phosphorylated protein assessed using membrane densitometry. Three independent experiments were performed. *p<0.05. (C) Significant inhibition of CXCL13-dependent *TAGLN2* KD Raji cell chemotaxis as compared with negative controls. *p<0.05. RFU, relative fluorescence units. Migratory cells were lysed and quantified using fluorescent dye. The relative quantification was used to determine the change between various samples. Data were normalized by designating one sample in negative controls as equal to 1. Then, the ratiometric results were used to scale all values relative to that sample. (D) The CXCR5 and IgM expression levels were assessed by flow cytometry in *TAGLN2* KD and control Raji cells. *TAGLN2* KD appeared to have no effect on the surface expression of CXCR5 and IgM. Gray shading indicates isotype control.

The actin cytoskeleton plays critical roles in both the initiation and regulation of BCR signaling [16,17]. To determine if TAGLN2 plays a role in actin cytoskeleton regulation in B-cell activation, Raji cells were transfected with scrambled or *TAGLN2* siRNA for 24 h and were then stimulated with10 μg/μL F(ab') anti-human IgM+IgG for 10 min. As shown in the immunoblots in Fig 4A, there was impaired phosphorylation of PLCγ2 that has been related to actin-linked signaling pathways [16] after IgM+IgG stimulation in *TAGLN2*-knockdown (KD) Raji cells compared to control cells. This result suggested that TAGLN2 may be an important actin regulator in B-cells. *TAGLN2* KD did not affect the levels of phosphorylated or total PI3K, ERK or Akt (Fig 4B).

Because *TAGLN2* KD in human breast cancer cells inhibited their migration and invasion [18], we performed the migration assay to examine whether TAGLN2 has a role of B cell migration. In the migration assay, fewer *TAGLN2* KD cells migrated to the lower chamber in a CXCL13-dependent manner than negative control cells (p<0.05), suggesting that TAGLN2 may have a role in cell migration (Fig 4C). *TAGLN2* KD did not change the expression levels of the cell surface molecules CXCR5 or BCR as determined by flow cytometry (Fig 4D).

### TAGLN2 is associated with the functional gene groups of the immune system including CCR6 and the regulation of the actin cytoskeleton

**Fig 5:**
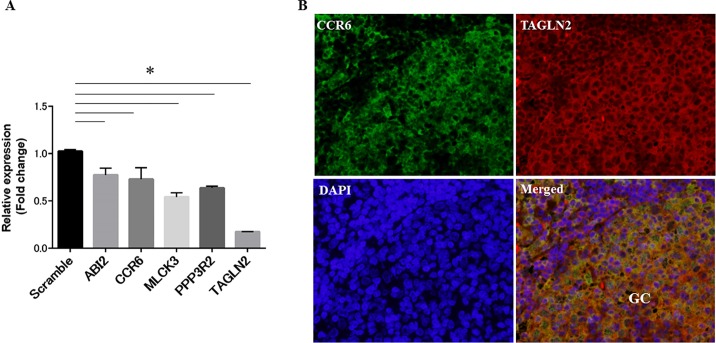
TAGLN2 is associated with the functional gene groups of the immune system including *CCR6* and the regulation of the actin cytoskeleton including *ABI2*. (A) Validation of the microarray data regarding the change in expression of specific genes in *TAGLN2*-knockdown Raji cells compared to control cells. Expression of the indicated genes was assessed using qRT-PCR and is indicated as fold change relative to that in control Raji cells transfected with scrambled siRNA. Results are the mean ± SEM of triplicate experiments. *p<0.05. (B) CCR6 is colocalized with TAGLN2^+^B-cells in GC areas of enlarged follicles in lymph nodes in SLE patients. Double immunofluorescence of CCR6 and TAGLN2 was performed. Nuclei were stained with DAPI. Co-localization of CCR6 and TAGLN2 is indicated in yellow in the merged image (original magnification, x400). GC, germinal center.

Next we tested whether *TAGLN2* KD affected intracellular BCR signaling events that were related to actin-linked signaling pathways. We compared the mRNA expression profiles of control cells and of Raji *TAGLN2* KD cells transfected with *TAGLN2* siRNA, following stimulation with IgM+IgG for 2 min, using Affymetrix microarray technology. This analysis showed a decrease in functional groups of the immune system in *TAGLN2* KD cells compared to the control (Table 1). Among the functional groupings of genes associated with the immune system, the chemokine (C_C motif) receptor 6 **(**CCR6) was chosen for further analysis because of its role in optimal GC reaction of activated B-cells [19,20] (Table 2). Abl-interactor 2 (*ABI2)* and myosin light chain kinase 3 (*MLCK3)*, which are in the pathway of the regulation of the actin cytoskeleton, and protein phosphatase 3, regulatory subunit B, beta (*PPP3R2)*, which is a downstream signaling molecule in the PLCγ-Ca2+-calcineurin pathway upon BCR signaling [21] (Table 3), were also selected and their expression in *TAGLN2*-knockdown and control Raji cells was validated using qRT-PCR (Fig 5A). Immunofluorescence staining showed colocalization of TAGLN2 and CCR6 in the area of GC in the enlarged follicles of lymph nodes in SLE and tonsils, suggesting that both proteins were expressed on activated B-cells (Fig 5B).

**Table 1:** Pathway analysis of microarray data. Functional grouping of significant, differentially expressed down regulated genes in *TAGLN2* siRNA Raji cells versus the control (p<0.01).

Pathway Name	Changed Genes	P-value
Signaling by GPCR	78	0.000002
GPCR downstream signaling	71	0.000002
GPCR ligand binding	39	0.00004
Cell Cycle, Mitotic	3	0.00034
Gene Expression	25	0.00065
Disease	19	0.00098
Immune System	27	0.00132
Signaling by NGF	3	0.00187
Signaling by the B Cell Receptor (BCR)	1	0.00236
NGF signaling via TRKA from the plasma membrane	1	0.00241
DNA Replication	1	0.00244
Neurotransmitter Release Cycle	7	0.00274
Na+/Cl- dependent neurotransmitter transporters	5	0.00303
Transport of inorganic cations/anions and amino acids/oligopeptides	12	0.00315
Olfactory Signaling Pathway	30	0.00327
Class B/2 (Secretin family receptors)	11	0.00364
SLC-mediated transmembrane transport	23	0.00406
Peptide ligand-binding receptors	18	0.00429
Adaptive Immune System	14	0.00441
Antigen processing: Ubiquitination amp; Proteasome degradation	2	0.00635
Signal Transduction	99	0.00646
Downstream Signaling Events Of B Cell Receptor (BCR)	1	0.00722
Olfactory transduction	31	0.00780
Class A/1 (Rhodopsin-like receptors)	25	0.00808
Transport of glucose and other sugars, bile salts and organic acids, metal ions and amine compounds	11	0.00827
	

GPCR, G-protein-coupled receptor; NGF, nerve growth factor

**Table 2:** Down-regulated genes related to the immune system.

Fold change	Gene Symbol	Gene Description	Probe Set ID
0.477	KIR3DL2	killer cell immunoglobulin-like receptor, three domains, long cytoplasmic tail, 2	16865530
0.557	CSH1 // CSHL1 // GH1	chorionic somatomammotropin hormone 1 (placental lactogen) // chorionic somatomammotropin hormone-like 1 // growth hormone 1	16847686
0.595	IFNA16	interferon, alpha 16	17092835
0.608	LILRB4	leukocyte immunoglobulin-like receptor, subfamily B (with TM and ITIM domains), member 4	16865424
0.621	CD8A	CD8a molecule	16899928
0.624	JAK3	Janus kinase 3	16870274
0.641	FCGR1B	Fc fragment of IgG, high affinity Ib, receptor (CD64)	16692577
0.651	HLA-DRB1	major histocompatibility complex, class II, DR beta 1	17017900
0.659	SIRPB1	signal-regulatory protein beta 1	16916492
0.660	FBXO4	F-box protein 4	16984347
0.665	MRC1	mannose receptor, C type 1	16702881
0.672	KIR2DL2	killer cell immunoglobulin-like receptor, two domains, long cytoplasmic tail, 2	16876242
0.675	MAPK11	mitogen-activated protein kinase 11	16936432
0.690	FOS	FBJ murine osteosarcoma viral oncogene homolog	16786587
0.692	MRC1	mannose receptor, C type 1	16702836
0.692	DEFB113	defensin, beta 113	17019916
0.694	ULBP2	UL16 binding protein 2	17013650
0.705	**CCR6**	**chemokine (C-C motif) receptor 6**	17014651
0.714	KIR2DS5	killer cell immunoglobulin-like receptor, two domains, short cytoplasmic tail, 5	16876269
0.716	CTSK	cathepsin K	16692846
0.720	CARD9 // DNLZ	caspase recruitment domain family, member 9 // DNL-type zinc finger	17099901
0.720	KLRC2	killer cell lectin-like receptor subfamily C, member 2	16761359
0.733	IFI27	interferon, alpha-inducible protein 27	16787814
0.735	PSMB2	proteasome (prosome, macropain) subunit, beta type, 2	16685139
0.735	CD160	CD160 molecule	16692158
0.738	C7	complement component 7	16984304
0.741	LILRB5	leukocyte immunoglobulin-like receptor, subfamily B (with TM and ITIM domains), member 5	16875387
0.749	DEFA6	defensin, alpha 6, Paneth cell-specific	17074301

Fold change, gene expression in TAGLN2 knockdown cells divided by its expression in control cells

**Table 3:** Top 30 genes downregulated in TAGLN2 knockdown versus control cells.

Fold change	Gene Symbol	Gene Description	Probe Set ID
0.435	LIG3	ligase III, DNA, ATP-dependent	16833292
0.477	KIR3DL2	killer cell immunoglobulin-like receptor, three domains, long cytoplasmic tail, 2	16865530
0.496	ND6	NADH dehydrogenase, subunit 6 (complex I)	17100697
0.502	TRPM5	transient receptor potential cation channel, subfamily M, member 5	16734441
0.514	HPSE	heparanase	16977537
0.515	HPR	haptoglobin-related protein	16820947
0.517	PTGES	prostaglandin E synthase	17099076
0.526	OR52K2	olfactory receptor, family 52, subfamily K, member 2	16721166
0.526	OR4S2	olfactory receptor, family 4, subfamily S, member 2	16724734
0.528	IFNA13	interferon, alpha 13	17092862
0.533	WIPI1	WD repeat domain, phosphoinositide interacting 1	16848079
0.533	PHKG1	phosphorylase kinase, gamma 1 (muscle)	17058079
0.541	GJA5	gap junction protein, alpha 5, 40kDa	16692362
0.544	IDO1	indoleamine 2,3-dioxygenase 1	17068296
0.547	ACSM1	acyl-CoA synthetase medium-chain family member 1	16824655
0.551	**PPP3R2**	**protein phosphatase 3, regulatory subunit B, beta**	17096683
0.556	TLX1	T-cell leukemia homeobox 1	16708433
0.557	CSH1 // CSHL1 // GH1	chorionic somatomammotropin hormone 1 (placental lactogen) // chorionic somatomammotropin hormone-like 1 // growth hormone 1	16847686
0.559	SLC16A3	solute carrier family 16 (monocarboxylate transporter), member 3	16839019
0.566	ORAI3	ORAI calcium release-activated calcium modulator 3	16818083
0.569	PNLIPRP2	pancreatic lipase-related protein 2	16709673
0.572	RAMP3	receptor (G protein-coupled) activity modifying protein 3	17045787
0.574	PRPH	peripherin	16750743
0.577	HIST1H4F	histone cluster 1, H4f	17005596
0.577	OR10P1	olfactory receptor, family 10, subfamily P, member 1	16752183
0.580	CLDN4	claudin 4	17046982
0.580	**ABI2**	**abl-interactor 2**	16889779
0.584	TNFRSF9	tumor necrosis factor receptor superfamily, member 9	16681288
0.586	**MYLK3**	**myosin light chain kinase 3**	16826195
0.587	ALPPL2	alkaline phosphatase, placental-like 2	16892246

## Discussion

The actin cytoskeleton is an important factor in the regulation of BCR activation [16,17]. Actin acts as a scaffold for the clustering of proteins, drives their centripetal translocation and organizes these microclusters to different domains to form the immunological synapse [22]. TAGLN2 is an actin-binding protein that stabilizes F-actin [2]. In our imaging studies, F-actin and TAGLN2 colocalized in Raji cells and both proteins were depleted from the central area of the cell, and they moved to the cell periphery together, after stimulation. The cell central area is where BCR microclusters may accumulate to form a mature immunological synapse. *TAGLN2* KD attenuated actin-linked signaling pathways that involve phosphorylated PLCγ2 in Raji cells, and this effect is probably associated with an unstable actin structure at the immunological synapse [2]. This role of *TAGLN2* in actin-linked signaling pathways may also be connected to the observed down-regulation of CCR6 in stimulated *TAGLN2* KD Raji cells compared to stimulated control cells. CCR6 is expressed at a high level in activated B-cells that enter into the follicle and interact with T-cells, leading to an optimal GC response and high-affinity antibody production [19,20], which suggests that TAGLN2 may enhance B-cell activation. CCR6 is also essential for appropriate anatomical positioning of memory B-cells and for the ability of memory B-cells to be recalled to their cognate antigens [23]. Another gene that is down regulated in stimulated *TAGLN2* KD Raji cells compared to stimulated control cells was *PPP3R2*. This gene encodes calcineurin B, which is the regulatory subunit of calcineurin [24], and is a downstream signaling molecule in the PLCγ-Ca2+-calcineurin pathway upon BCR signaling [21]. PP3R2 activates NFAT and NFAT function depends on actin [21,25]. Of particular interest in terms of the present study is the fact that the calcineurin inhibitors have been widely used as immunosuppressive agents in transplantation and have been used as potential therapeutic agents in patients with lupus nephritis [26, 27].

Actin polymerization and depolymerization are regulated by accessory proteins including Arp2/3, WASp, WAVE, and cofilin [16]. TAGLN2 competes with cofilin for binding to F-actin and blocks cofilin-mediated depolymerization in T-cells [2]. Down-regulation of *MLCK3* and *ABI2* in *TAGLN2* KD cells versus control cells was observed by microarray analysis. Abl interactor 2 recruits the WAVE2 regulatory complex to the plasma membrane and promotes actin polymerization and immunological synapse formation in T-cells [28] or in human cytomegalovirus-specific immune responses [29]. A previous report showed that TAGLN2 directly regulates myosin light chain phosphorylation and its total expression in endothelial cells [30]. Myosin light chain phosphorylation is mediated by myosin light chain kinase. Consistent with these reports, the migration of *TAGLN2* KD cells was inhibited versus control cells in a migration assay in our study. The overexpression of TAGLN2 in various malignant tumors and its enhancement of tumor migration and invasion have been reported [3,4,5,18]. These data suggest that TAGLN2 may be involved in actin-myosin contraction and cell migration in B-cells.

The combined T- and B-cell abnormalities in SLE result in the production of pathogenic autoantibodies. The pathogenic B-cell autoantibodies are high-affinity, somatically mutated and Ig-switched, which are the products of GC responses [10,11]. It was previously reported that members of the mitogen activated protein kinase family (MAPKs) were elevated in peripheral B-cells from SLE patients [31]. Increased phospho-Akt in SLE B-cells after B-cell ligation has also been reported [32]. These findings suggest inflammation-mediated hyperactivity of B-cells in SLE. Active SLE shows over-reactive T-cell-dependent GC reactions that produce expanded and largely unregulated numbers of memory B-cells and plasma cells [10]. Reactive follicular hyperplasia is the most frequent finding in the lymph node in SLE patients, which reflects intense GC activity [12]. TAGLN2^+^B-cells were distributed from the follicular/GC to perifollicular areas, where activated GC B-cells and memory B-cells, respectively, are located [19,20]. CCR6 has been shown to be important for antigen-driven B-cell differentiation, which is seen in GC B-cells and memory B-cells [19,20], and the colocalization of TAGLN2 and CCR6 from the area of the GC to the perifollicular area was observed in SLE lymphadenopathy. TAGLN2 expressed in activated B-cells was localized from follicular/GC areas to perifollicular areas in secondary lymphoid tissues in SLE. Our study of primary healthy B-cell cultures confirmed that *TAGLN2* mRNA can be induced by IgM+IgG stimulation within 24 h. Following B-cell activation through the BCR, activated B-cells can enter the GC and differentiate into plasma cells or memory B-cells [17]. There was an increase in TAGLN2 expression in B-cells in the kidney in SLE patients, where ectopic lymphoid structures are found at sites of chronic inflammation [33]. In target organs including kidneys in SLE, organized accumulations of T- and B-cells resembles secondary lymphoid organs and generate autoreactive antibodies [33], although the renal biopsy specimens were too small to assess the structures in our study. However, *TAGLN2* mRNA expression in peripheral blood CD19^+^ B-cells as well as in CD19^+^CD27^+^ memory B-cells was not different between healthy donors and SLE patients. The ratios of CD38^+^ B-cells among CD19^+^CD27^+^ memory B-cells and CD19^+^ B-cells tended to increase in SLE patients, suggesting peripheral B-cells are in the activation state in SLE patients. Although data were unavailable for CD69 expression, the early activation marker of B-cells, to assess the activation state of B-cells in our study [17], our results suggested that the expression levels of TAGLN2 might decrease after activated B-cells differentiate into plasma cells and memory B-cells and enter the peripheral circulation. However, there is a possibility that the fact that our patients were given steroid might have had an undesirable influence on the results of peripheral B-cells.

In conclusion, TAGLN2 induced the formation of the actin cytoskeleton and TAGLN2^+^B-cells were distributed from the germinal center to perifollicular areas, which reflected activated B-cells in SLE with intense GC formation. TAGLN2 may regulate B-cell activation in secondary lymphoid tissues and could be used as a marker for activated B-cells.

## Supporting information

S1 Fig To assess B-cell activation, CD38 expression was assessed by flow cytometry. The ratio of CD38^+^/CD19^+^ B-cells in SLE (n = 5) and the controls (n = 9) was 81 ± 1.8% and 88 **±** 2.5%, respectively (p = 0.05). Further, the ratio of CD38^+^/CD19^+^CD27^+^ B-cells in SLE (n = 5) and the controls (n = 9) was 71 **±** 7.3% and 55 ± 2.6%, respectively (*p<0.05).
(TIF)

S1 Table The details of the relative *TAGLN2* mRNA expression levels and the ratio of CD38^+^ cells among CD19^+^ B-cells or CD19^+^CD27^+^ B-cells in SLE patients and controls. (DOCX)

S1 Video Corresponding to Fig 3 at time 0, shows localization of actin (LifeAct-RFP) and TAGLN2-GFP in Raji cells before IgM+IgG stimulation. (AVI)

S2 Video Corresponding to Fig 3, shows both the actin and TAGLN2 signals increased in intensity and thickness after IgM+IgG stimulation. (AVI)

## Abbreviations

TAGLN2: Transgelin-2
BCR: B-cell receptor
SLE: systemic lupus erythematosus
GC: germinal center
qRT-PCR: quantitative real-time PCR
PBMCs: peripheral blood mononuclear cells
SEM: standard error of the mean
KD: knockdown
