# Supplementary figures and images for: Transgelin-2 is upregulated on activated B-cells and expressed in hyperplastic follicles in lupus erythematosus patients
Source: PLoS One. 2017 Sep 14;12(9):e0184738. doi: 10.1371/journal.pone.0184738 (PMC5599031; doi:10.1371/journal.pone.0184738)

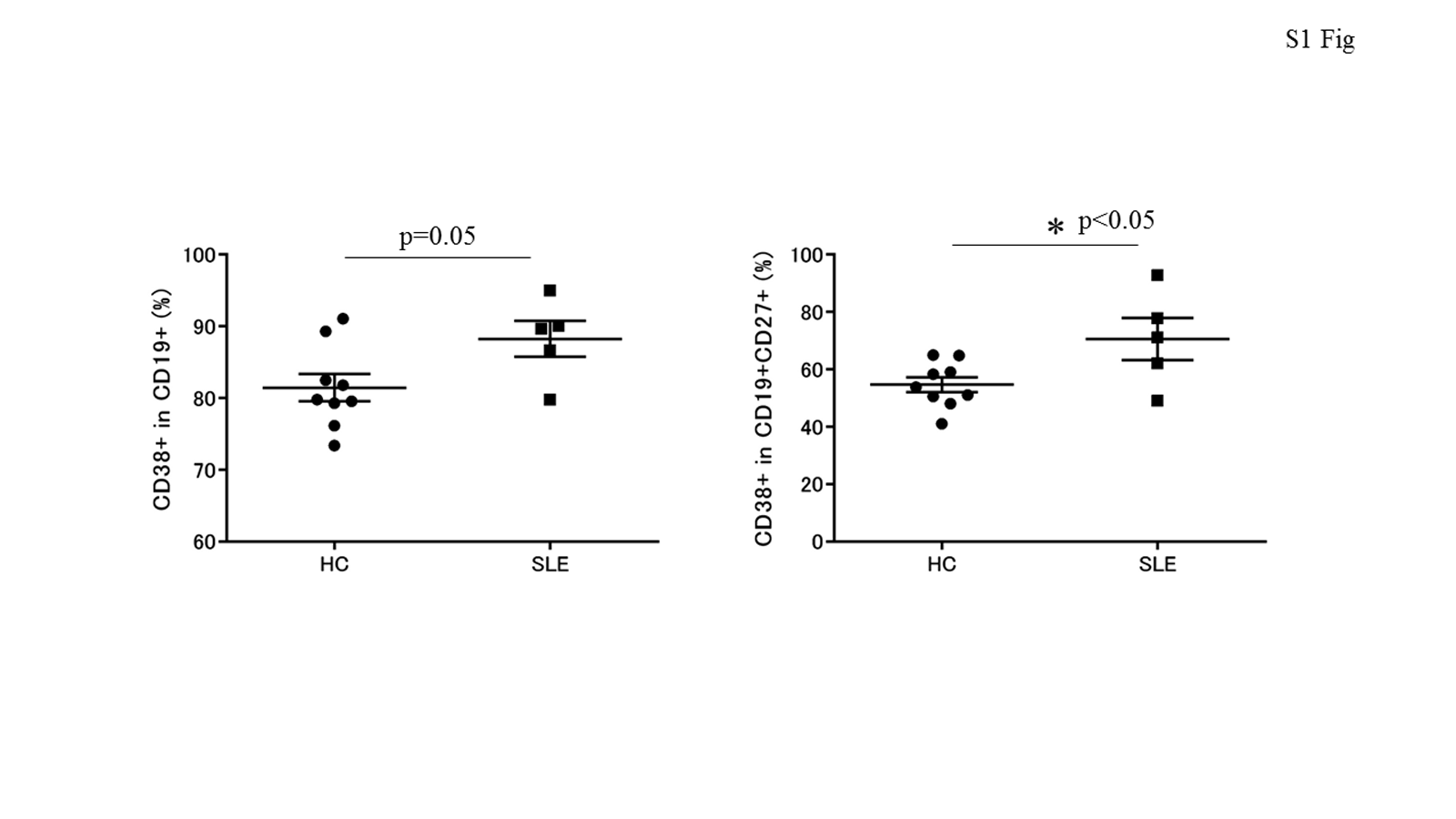

Supplement: S1 Fig — The ratio of CD38+/CD19+ B-cells in SLE (n = 5) and the controls (n = 9) was 81 ± 1.8% and 88 ± 2.5%, respectively (p = 0.05). Further, the ratio of CD38+/CD19+CD27+ B-cells in SLE (n = 5) and the controls (n = 9) was 71 ± 7.3% and 55 ± 2.6%, respectively (*p<0.05). (TIF) [file pone.0184738.s001.tif]
