# Supplementary material for: Transgelin-2 is upregulated on activated B-cells and expressed in hyperplastic follicles in lupus erythematosus patients
Source: PLoS One. 2017 Sep 14;12(9):e0184738. doi: 10.1371/journal.pone.0184738 (PMC5599031; doi:10.1371/journal.pone.0184738)
Supplement: S1 Table — (DOCX) [file pone.0184738.s002.docx]

S1_Table: The levels of *TAGLN2* mRNA and CD38 expressions in peripheral B cells of SLE patients and healthy controls

| **Patient** | **Relative *TAGLN2* mRNA expression in CD19+ B cells (fold change)** | **Relative *TAGLN2* mRNA expression in CD19+CD27+ B cells (fold change)** | **CD38 expression in CD19+ B cells (%)** | **CD38 expression in CD19+CD27+ B cells (%)** |
| --- | --- | --- | --- | --- |
| SLE71 | 0.52497 | 1.25197 | 86.7 | 92.8 |
| SLE64 | 0.70406 | 0.43075 | 95 | 77.9 |
| SLE51 | na | 0.81459 | 89.7 | 62.2 |
| SLE47 | 0.73247 | 1.08807 | 79.8 | 71.1 |
| SLE45 | 0.94809 | 0.49115 | 90.1 | 49.1 |
| SLE39 | na | 1.6868 | na | na |
| SLE38 | na | 2.03557 | na | na |
| SLE37 | 0.87054 | 1.20316 | na | na |
| SLE36 | 0.83600 | na | na | na |
| SLE31 | 0.73852 | 1.39071 | na | na |
| SLE29 | na | 2.19051 | na | na |
| SLE28 | 0.79614 | na | na | na |
| SLE25 | na | 1.04031 | na | na |
| SLE24 | na | 0.8833 | na | na |
| SLE23 | 0.76556 | na | na | na |
| SLE16 | na | 1.43818 | na | na |
| SLE1 | na | 0.7423 | na | na |
| **Controls** |  |  |  |  |
| HC36 | na | 1.44175 | 91.1 | 65 |
| HC35 | 1.22758 | 1.06783 | 89.3 | 53.9 |
| HC34 | 1.05828 | 1.1061 | 82.5 | 64.9 |
| HC33 | na | 0.80701 | 79.8 | 59.1 |
| HC32 | 0.57553 | 0.57617 | 76.2 | 58.4 |
| HC31 | 1.16712 | 0.83976 | 73.4 | 48.1 |
| HC30 | na | 0.38059 | 79.3 | 50.6 |
| HC27 | na | 0.88867 | 79.6 | 41.1 |
| HC16 | na | 1.19066 | 81.8 | 51.1 |
| HC15 | 1.12904 | na | na | na |
| HC12 | na | 1.67549 | na | na |
| HC03 | 0.78303 | 1.02596 | na | na |

na, not available
